# The Effect of Leisure Activity Diversity and Exercise Time on the Prevention of Depression in the Middle-Aged and Elderly Residents of Taiwan

**DOI:** 10.3390/ijerph15040654

**Published:** 2018-04-01

**Authors:** Hsiao-Yun Lee, Chia-Pin Yu, Chih-Da Wu, Wen-Chi Pan

**Affiliations:** 1 School of Forestry and Resource Conservation, National Taiwan University, Taipei 106, Taiwan; leehsi@ntu.edu.tw; 2 Department of Forestry and Nature Resources, National Chiayi University, Chiayi 600, Taiwan; chidawu@mail.ncyu.edu.tw; 3 Institute of Environmental and Occupational Health Sciences, National Yang-Ming University, Taipei 112, Taiwan; wenchipan@ym.edu.tw

**Keywords:** leisure activity diversity, exercise, middle-aged and elderly, depression, CESD-10

## Abstract

Previous studies have confirmed that activity participation is beneficial to mental health, but few studies focus on older adults’ depression. Based on the theory of social integration, this study examined the effects of leisure activity diversity and exercise time on depression in late adulthood. Subjects in the 2011 Survey of Health and Living Status of the Middle-Aged and Elderly in Taiwan were extracted. A series of logistic regressions were conducted to discern factors related to the odds of having depression. Among study subjects (*N* = 3727; age ≥ 58), 20.9% indicated an inclination of having depression (CESD-10 score ≥ 8). This study found that participating in diverse leisure activities and longer exercise time decreases older adults’ risk of having depression. Additionally, the results confirmed that depression is positively correlated with chronic diseases. Consequently, efforts should be continually spent on encouraging older adults’ participation in activities to reduce the prevalence of depression.

## 1. Introduction

Evidence from previous studies has shown that physical activity participation is negatively associated with depression. Blake et al. [1] reviewed eleven studies and concluded that those who engage in the exercise had a short-term positive outcome for alleviating depressive symptoms. Similar results are revealed by other studies [2,3,4] confirming exercise as an alternative therapy in reducing depression and anxiety. Mammen and Faulkner [5] found that even conducting lower-intensity exercise (e.g., walking) might prevent the occurrence of depression. In addition to exercise, the association between leisure activity participation and mental well-being has also been examined. Consistent results were reported by previous studies that leisure activity engagement is related to better mental well-being and life satisfaction [6,7]. Fine [8] concludes that participating in leisure activity, including sedentary leisure activity (e.g., card games), was positively associated with mental health in the elderly. 

Despite substantial evidence demonstrating the benefit of activity participation on mental health, few studies focus on depression in late adulthood. The prevalence rate of depression among older adults has been observed and proved to be a significant indicator of physical and mental health in this age group. According to a statistical report conducted in the United States, about 1–5% of older adults in a community setting are suffering from depression [9,10]. In Taiwan, it is estimated that 13–21% of older adults have depression, and the trend is increasing. Both reports illustrate the high prevalence rate of depression among the aging population [11]. Depression not only reduces general health [12] and quality of life [13], it also increases medical costs [14] and the odds of suicide [15]. Chong et al. [16], in a study conducted in Taiwan, reported that older adults have the highest suicide rates of all age groups, and one of the main reasons for their suicide is depression. Although the causes of depression are complicated, non-genetic factors may be prevented or alleviated by engaging in healthy behaviors.

According to the social integration perspective, engaging in diverse activities (e.g., household chores, paid work, and leisure activity) can enhance individuals’ social networks and knowledge, which are fundamental elements of sound mental well-being [17,18]. Correspondingly, it can be assumed that participating in a wide range of leisure activities may be a protective factor against depression in older adults. Nonetheless, there is a paucity of information about the effect of diverse leisure activities on older adults’ mental health; it is not yet known if diverse leisure activities is as beneficial as exercise in combating older adults’ depression. As a result, the aim of this study is to examine whether or not leisure activity diversity and exercise time are both protective factors against depression in late adulthood. Furthermore, differences between gender and age groups, which can contribute to differences in managing life, are also considered. Because this study uses nationally representative data, the results are expected to provide insightful information about preventive methods for older adults’ depression; consequently, policy recommendations could be made based on the findings.

## 2. Materials and Methods

### 2.1. Data and Study Sample

Data were extracted from the 2011 Survey of Health and Living Status of the Middle-Aged and Elderly in Taiwan, conducted by the Ministry of Health and Welfare, Taipei, Taiwan [19]. This survey is comprised of a nationally representative sample, focusing on older adults. Besides demographic information, the data included information related to family structure, health condition, healthcare utilization, leisure activity, welfare utilization, etc. Detailed information about the survey design can be found in previous studies [20,21].

### 2.2. Measurement

The outcome variable was the subjects’ depression symptoms discerned by adopting CESD-10 [22,23,24]. CESD-10 has been proven to have comparable validity and accuracy to CESD-20 [23]. The score range of CESD-10 is from 0 to 30. A higher score illustrates more severe depression symptoms. The cutoff ≥8 score was adapted in this study [23]. Subjects who scored 8 points or higher indicated an inclination toward depression.

Demographic variables included gender (male/female), age, education (primary school or lower/secondary school/senior high school/college or higher), having a partner (i.e., spouse or companion), and satisfaction with economic status. The subjects’ satisfaction with economic status was measured using a 5-point Likert scale. A higher score illustrated a higher satisfaction with economic status. Cigarettes and alcohol, the most popular substances in Taiwan [25], were investigated to reflect the subjects’ substance use. Two questions adapted from the Behavioral Risk Factor Surveillance System (BRFSS) were asked to investigate the subjects’ smoking statuses [26]. Subjects who had smoked 100 cigarettes (5 packs) or more in their entire life and who currently smoke every day or some days were categorized as cigarette users. Otherwise, they were categorized as nonusers. The subjects’ drinking statuses were discerned by one question: “Did you use any alcohol in the past year?” Subjects who indicated they had used any kind of alcohol were categorized as alcohol users. Three common chronic diseases of older adults were chosen to reflect the subjects’ health statuses, including hypertension, diabetes, and cardiovascular disease. Each disease was dichotomized as present or not. Other than diseases, body mass index (BMI) was also applied to discern health status, calculated by the subjects’ height and weight information.

Regarding leisure activity participation, subjects were asked if they participated in the following twelve activities: (1) watching television, (2) listening to music or radio, (3) reading newspapers or books, (4) web surfing, (5) playing games such as chess or mahjong, (6) meeting with friends, (7) meeting with neighbors, (8) gardening, (9) talking a walk, (10) cycling, (11) jogging, and (12) joining group activities. Subjects scored 1 for participating in each activity. A higher score indicated participation in more activities, reflecting their leisure activity diversity. The subjects’ total exercise times per week were calculated by two questions: (1) How often do you exercise in a week? and (2) How many minutes do you exercise each time? The subjects’ total exercise times were calculated by multiplying exercise frequency by exercise duration.

### 2.3. Statistical Analysis

A series of logistic regression analyses were conducted to capture the influences of variables on older adults’ depression, considering the differences between gender and age. Model 1 includes all of the population, while model 2 and model 3 have only single gender subjects. Model 4 includes middle-aged subjects (ages 58–64), while model 5 includes elderly subjects (age 65 or older). All statistical analyses were conducted using Stata^®^ 14.2. (StataCorp, College Station, TX, USA), and all estimates in regression models were weighted based on the sampling scheme.

## 3. Results

The results of the descriptive analysis are shown in Table 1. Of all the subjects (*N* = 3727), 48.1% are male (*N* = 1793), and 51.9% are female (*N* = 1934). The average age is 69.04 (SD: 8.86). The majority have an education level of primary school or lower (66.3%), a spouse or a companion (68.3%), and an income satisfaction level above the average (Mean: 3.21; SD: 0.93). Regarding substance use, 13.4% currently use cigarettes, and 29.3% use alcohol. In terms of health status, nearly half of the subjects have hypertension (45.4%), while fewer have diabetes (19.6%) and cardiovascular disease (18.8%). The average BMI is 24.25 (SD: 3.52). With respect to the subjects’ health behavior, the mean of leisure activity diversity is 5.01 (SD: 2.34), and the mean of the total exercise time in one week is 162.93 min (SD: 166.65). Lastly, 20.9% of the subjects reported an inclination to having depression (Mean: 13.64; SD: 5.09). 

The results of a logistic regression model of the study population are also shown in Model 1. Odds ratios (ORs) illustrate the relative odds of having depression. Being a man (OR = 0.73, 95% confidence interval (CI) 0.55–0.97), having higher income satisfaction (OR = 0.49, 95% CI 0.43–0.56), having a partner (OR = 0.56, 95% CI 0.43–0.73), and using alcohol (OR = 0.67, 95% CI 0.49–0.91) decrease the odds of having depression. In contrast, having chronic diseases increases the odds of having depression (hypertension: OR = 1.60, 95% CI 1.23–2.07; diabetes: OR = 1.50, 95% CI 1.13–1.99; cardiovascular disease: OR = 1.70, 95% CI 1.28–2.26). Lastly, an increase of one unit in BMI (OR = 0.93, 95% CI 0.90–0.97), leisure activity diversity (OR = 0.81, 95% CI 0.76–0.87), and total exercise time (OR = 0.99, 95% CI 0.99–0.99) decreases the subjects’ odds of having depression.

Table 2 shows the results of logistic regression analyses by gender and age, respectively. Model 2 includes only male subjects and shows that predictors such as having higher income satisfaction (OR = 0.50, 95% CI 0.42–0.60), having a partner (OR = 0.46, 95% CI 0.30–0.70), and using alcohol (OR = 0.57, 95% CI 0.39–0.82) are negatively related to depression. Among the three chronic diseases, only having hypertension increases the odds of having depression (OR = 1.69, 95% CI 1.14–2.48). Lastly, a one-unit increase in BMI (OR = 0.92, 95% CI 0.88–0.98) and leisure activity diversity (OR = 0.78, 95% CI 0.70–0.86) decreases the subjects’ odds of having depression. In the female-only model (Model 3), the results show that having higher income satisfaction (OR = 0.49, 95% CI 0.41–0.92) and having a partner (OR = 0.65, 95% CI 0.46–2.88) decrease the odds of having depression. In contrast, age (OR = 1.03, 95% CI 1.00–1.05) and chronic diseases are found to be positively related to having depression (hypertension: OR = 1.55, 95% CI 1.09–2.19; diabetes: OR = 1.72, 95% CI 1.16–2.55; cardiovascular disease: OR = 1.91, 95% CI 1.27–2.86). Lastly, a one-unit increase in BMI (OR = 0.94, 95% CI 0.89–0.98), leisure activity diversity (OR = 0.85, 95% CI 0.76–0.94), and total exercise time (OR = 0.99, 95% CI 1.00–1.00) are found to be negatively associated with depression.

Model 4 includes middle-aged subjects (ages 58–64), and the results show that higher income satisfaction (OR = 0.44, 95% CI 0.36–0.70) and having a partner (OR = 0.44, 95% CI 0.28–2.88) decrease the odds of having depression. Having hypertension (OR = 1.91, 95% CI 1.24–2.95) and cardiovascular disease (OR = 2.23, 95% CI 1.31–3.78), in contrast, are found to increase the subjects’ odds of having depression. Lastly, a one-unit increase in BMI (OR = 0.92, 95% CI 0.86–0.98) and leisure activity diversity (OR = 0.78, 95% CI 0.70–0.88) decrease the odds of having depression. Model 5 includes elderly subjects (age 65 and older), and the results show that being a man (OR = 0.66, 95% CI 0.47–0.92), having higher income satisfaction (OR = 0.55, 95% CI 0.47–0.65), having a partner (OR = 0.62, 95% CI 0.45–0.84), and using alcohol (OR = 0.66, 95% CI 0.45–0.97) are negatively associated with depression. Moreover, a one-unit increase in BMI (OR = 0.94, 95% CI 0.90–0.98), leisure activity diversity (OR = 0.84, 95% CI 0.77–0.92), and total exercise time (OR = 0.99, 95% CI 1.00–1.00) decrease the odds of having depression. In contrast, having a chronic disease (hypertension: OR = 1.43, 95% CI 1.05–1.96; diabetes: OR = 1.44, 95% CI 1.02–2.03; cardiovascular disease: OR = 1.50, 95% CI 1.08–2.09) increases the odds of having depression.

## 4. Discussion

### 4.1. The Influence of Leisure Activity Diversity and Exercise Time on Depression

Previous studies have revealed that leisure activity participation can improve psychological well-being in older adults [7,27,28]. Similarly, our study results show that participating in leisure activities is associated with lower odds of having depression in older adults. Furthermore, we found that the more diverse leisure activities older adults attended, the lower their risk of depression. This finding may be explained by social integration theory, proposed by Durkheim in 1897 and then broadly applied in the health field by other researchers [29]. Berkman et al. [29] present a conceptual model illustrating how social networks influence personal health. Within the model, social networks provide opportunities for individuals to gain social support, engage in social activity, and be influenced with respect to their health behaviors. Subsequently, the above-mentioned mechanisms influence individuals’ health behavior and psychological states, including self-efficacy and self-esteem, which are protective factors against depression. Accordingly, engaging in diverse leisure activities, such as meeting with friends and joining group activities, provides older adults opportunities to gain social support, leading to better mental health.

The beneficial effect of exercise on depression prevention has been confirmed in the general population [5]. However, the effect of exercise on older adults’ depression was not consistent [30,31]. Our results show that increasing exercise time decreases the risk of depression for female and elderly subjects. Our finding is in line with some previous studies [32,33,34]; nonetheless, the rationale underlying the different results between gender and age groups is not yet clear. Hsiao, who investigated the exercise behavior of 900 older adults in Taiwan [35], indicated that the main purpose of exercise for this age group was health and fun. Therefore, we hypothesized that the different results for gender and age may have occurred because women and elderly are more likely to be satisfied by exercise in terms of having fun and enjoying the health benefits, leading to a lower risk of depression. In addition to exercise expectations, the different effects on gender and age groups may be rooted in types of exercise. Older adults who more often participated in group-orientated exercises would have more opportunities for gaining social support—thereby decreasing their chances of depression—than those who primarily participated in individual-orientated exercises. Given that there is a paucity of information about exercise types and subgroups, more studies should be conducted in the future to confirm the rationale underlying the different effects of exercise on different subgroups.

### 4.2. Demographic Differences in Depression

Consistent with the majority of the previous studies [10,36,37,38], our results show the gender difference in depression. To be more detailed, older women were found to have higher odds of having depression than their male counterparts. Mamplekou et al. [38] suggest that older women’s mental health difficulties might be related to lower income. This suggestion may partially explain why older Taiwanese women are more likely to experience depression than men. In Taiwan, it is generally believed that men should provide financial support while women should be fully responsible for household chores [39]. Without work experience and ability, the majority of older women lose their income when their husbands die, which may contribute to depression. Moreover, this division of gender roles deprives women of opportunities to engage in social activities, which is crucial for preventing depression. Norton et al. [37], however, point out that gender differences in the prevalence rate of depression might be overestimated because male gender and depression are both positively related to mortality. In other words, the lower prevalence rate of depression among men may be due to their shorter lifespans. Therefore, it is suggested that postmortem information should be considered in future studies to precisely capture the gender differences in depression.

Results of this study also show that having a life partner is a protective factor against depression for older adults, consistent with previous studies [10,40,41]. Some researchers claim that this is because having a partner provides more opportunities for close social contact and emotional support [42], both essential to mental health. Consequently, being single, separated, or widowed would increase the likelihood of depression due to social isolation [42,43,44]. Kessler and Essex [45] provide different perspectives and report that married people are more resilient and better able to cope with stress than non-married people. In addition, they indicate that individuals with mental illness have fewer opportunities to get married, leading to the difference in depression prevalence.

There is no doubt that income plays a significant role in predicting older adults’ depression [10,40,46]. Our findings show that lower income satisfaction increases the likelihood of having depression across both gender and age groups. Rationales underlying the relationship between income and depression are complicated. Some researchers claim that lower income could result from involuntary job loss [47]. The impact of job loss can lead to decreased social connections and increased social isolation, thereby increasing the odds of having depression. Other researchers have hypothesized that medical access may be the mediator causing the disparity [40]. Those with higher incomes have superior medical resources and are able to afford psychiatric treatment, resulting in lower depression prevalence rates. Given that Taiwan has employed a universal health care system for twenty years, it is not yet clear whether or not patients’ access to or quality of treatment is related to the disparity in depression. Therefore, more studies are needed to investigate financial determinants influencing the prevalence of depression.

### 4.3. The Influence of Substance Use and Health Status on Depression

Substance abuse has been a confirmed correlate with mental disorders [48,49]. Nonetheless, the association between moderate substance use and depression is rarely examined. Our results show that any alcohol use in the past year decreased older adults’ odds of having depression. This finding is not consistent with previous studies [50,51,52]. However, the negative relationship between alcohol consumption and depression is only observed in the male group and the elderly group, indicating behavioral differences across gender and age groups. The positive effects of drinking for men may result from their involvement in social events, which are beneficial to their mental health. In contrast, drinking has not been considered an appropriate behavior for women in Taiwan in the past [53]. Hence, the effect of drinking on women might not be as advantageous as it on men. Regarding age differences, study results showed that alcohol use is negatively related to depression among the elderly but not among middle-aged adults. The different effects across age groups might result from the fact that most middle-aged adults in Taiwan are still at work; part of their alcohol use could be associated with stressful job-related activities. In contrast, given that most of the elderly population is retired, their alcohol use may be related to informal social activities, which have been confirmed to significantly reduce the odds of having depression [46].

There is substantial evidence showing that chronic disease is a strong predictor for older adults’ depression. In line with previous studies [40,41,54], our results show that having a chronic disease (i.e., hypertension, diabetes, or cardiovascular disease) increases older adults’ odds of having depression, confirming the positive relationship between physical illness and depression. A few studies further discern how physical illness influences older adults’ depression. Mojtabai and Olfson [40] propose that the biological element of physical illness might cause depression. Other than the biological mechanism, we suggest for consideration the stressors derived from chronic diseases, such as long-term management of disease and the financial burden of medical costs. Those persistent stressors could increase the risk of depression among older adults. Our study results show that BMI is negatively associated with depression across gender and age groups. Given that prior studies did not show consistent results regarding the association between BMI and depression [55,56,57,58], more studies are needed to supplement the literature.

### 4.4. Policy Implications and Limitations

This study examined determinants influencing older adults’ depression, focusing on the effects of diversity of leisure activity and exercise time. It provides substantial empirical data from a Taiwanese perspective. Further, it shows that disparities exist in the odds of having depression among older adults, allowing us to make policy implications based on the findings. First, older adults with lower income satisfaction are more likely to have depression. Hence, in addition to improving social welfare, greater efforts should be made to increase medical accessibility and affordability for older adults with low incomes. Eliminating difficulties in accessing medical care and removing financial barriers for medical treatments should improve equity of patient care. Second, chronic diseases are confirmed to have a strong positive relationship with depression. Other than educating older adults about how to manage chronic diseases, actions should be taken to teach the population how to prevent chronic diseases at an earlier stage, given that most chronic diseases are preventable by adopting a healthy lifestyle. Lastly, participating in diverse leisure activities and engaging in physical activity is associated with a reduced risk of having depression in late adulthood. Therefore, continuous efforts should be made to encourage participation in activities to reduce the prevalence rate of depression. Local government and communities should take responsibility for creating and maintaining an elderly-friendly environment for conducting activities.

This study has limitations. First, the secondhand data contains self-reported information, which may have issues such as recall bias. Second, the prevalence rate of depression changes over time. It is expected that some subjects died from depression. Nonetheless, this study does take postmortem information into account. Third, depression that results from life events, such as the death of a spouse, are not considered in this study. Lastly, it should be further noted that the cross-sectional design in the current study limits definitive conclusions about directionality. In other words, does activity participation cause reduced risk of depression, or vice versa in this study population? Perrino et al. [59] found that depression predicted their subjects’ future physical activity/inactivity. Therefore, issues of causality or directionality require careful prospective longitudinal study designs and analyses to further determine the directionality of the relationships between activity participation (both in leisure activities and exercise) and depressive symptomatology, over time.

## 5. Conclusions

In sum, disparities were found in the odds of having depression among older adults in Taiwan. Though there are predisposing factors (e.g., gender), changing one’s lifestyle by engaging in diverse leisure activities and physical activities can reduce the odds of having depression in older adults. Consequently, more effort should be spent on promoting activity participation as a method for preventing depression in older adults.

## Figures and Tables

**Table 1:** Descriptive statistics of study population and regression estimates of having depression.

Variable	*N* (%)/Mean (SD)		Model 1: Total Population		
			OR	t	95% CI
**1. Demographics**					
Male	1793	(48.1%)	0.73 *	−2.20	(0.55, 0.97)
Age	69.04	(8.86)	1.01	1.54	(1.00, 1.03)
Education level:					
Primary school or less	2470	(66.3%)	-		
Junior High school	423	(11.3%)	0.73	−1.65	(0.50, 1.06)
Senior High school	620	(16.6%)	0.96	−0.21	(0.69, 1.35)
College	215	(5.8%)	1.19	0.60	(0.67, 2.14)
Income satisfaction	3.21	(0.93)	0.49 ***	−10.75	(0.43, 0.56)
Partner	2545	(68.3%)	0.56 ***	−4.26	(0.43, 0.73)
**2. Substance use**					
Cigarettes	501	(13.4%)	0.83	−0.94	(0.56, 1.22)
Alcohol	1093	(29.3%)	0.67 *	−2.59	(0.49, 0.91)
**3. Health status**					
Chronic diseases:					
Hypertension	1694	(45.4%)	1.60 ***	3.56	(1.23, 2.07)
Diabetes	730	(19.6%)	1.50 **	2.82	(1.13, 1.99)
Cardiovascular disease	701	(18.8%)	1.70 ***	3.64	(1.28, 2.26)
BMI	24.25	(3.52)	0.93 ***	−3.82	(0.90, 0.97)
**4. Health behavior**					
Leisure activity diversity (Range: 0–12)	5.01	(2.34)	0.81 ***	−5.64	(0.76, 0.87)
Total exercise time (minute/week)	162.93	(166.65)	0.99 **	−3.19	(0.99, 0.99)
**5. Depression** (Range: 0–30; cutoff: 8)					
Yes	721	(20.9%)			
	13.64	(5.09)			
No	2725	(79.1%)			
	2.10	(2.22)			
	*N* = 3727				

* *p* <0.05; ** *p* <0.01; *** *p* <0.001. OR: Odds ratio; CI: Confidence interval.

**Table 2:** Regression estimates of having depression by gender and age.

Model: Subpopulation	Model 2: Male			Model 3: Female			Model 4: Middle-Aged			Model 5: Elderly		
Covariates	OR	t	95% CI	OR	t	95% CI	OR	t	95% CI	OR	t	95% CI
**1. Demographics**												
Male	-			-			0.93	-0.30	(0.58, 1.50)	0.66 *	−2.46	(0.47, 0.92)
Age	1.00	−0.36	(0.98, 1.02)	1.03 *	2.21	(1.00, 1.05)	-			-		
Education level:												
Primary school or less	-			-			-			-		
Junior High school	0.89	−0.42	(0.52, 1.52)	0.61	−1.77	(0.35, 1.06)	0.68	−1.16	(0.36, 1.30)	0.78	−1.01	(0.49, 1.26)
Senior High school	1.04	0.18	(0.66, 1.65)	1.00	<0.01	(0.60, 1.66)	1.18	0.64	(0.71, 1.99)	0.81	−0.90	(0.52, 1.28)
College	1.42	0.96	(0.70, 2.88)	1.00	<0.01	(0.35, 2.88)	0.97	−0.07	(0.40, 2.31)	1.52	1.03	(0.69, 3.34)
Income satisfaction	0.50 ***	−7.55	(0.42, 0.60)	0.49 ***	−7.61	(0.41, 0.92)	0.44 ***	−7.80	(0.36, 0.70)	0.55 ***	−6.93	(0.47, 0.65)
Partner	0.46 ***	−3.63	(0.30, 0.70)	0.65 *	−2.46	(0.46, 2.88)	0.44 **	−3.48	(0.28, 2.88)	0.62 **	−3.05	(0.45, 0.84)
**2. Substance use**												
Cigarettes	0.84	−0.85	(0.55, 1.26)	0.56	−1.02	(0.18, 1.71)	0.84	−0.58	(0.47, 1.51)	0.67	−1.44	(0.39, 1.16)
Alcohol	0.57 **	−3.05	(0.39, 0.82)	0.88	−0.48	(0.53, 1.47)	0.65	−1.66	(0.39, 1.08)	0.66 *	−2.10	(0.45, 0.97)
**3. Health status**												
Chronic diseases:												
Hypertension	1.69 **	2.64	(1.14, 2.48)	1.55 *	2.45	(1.09, 2.19)	1.91 **	2.94	(1.24, 2.95)	1.43 *	2.24	(1.05, 1.96)
Diabetes	1.30	1.23	(0.86, 1.97)	1.72 **	2.69	(1.16, 2.55)	1.56	1.73	(0.94, 2.57)	1.44 *	2.09	(1.02, 2.03)
Cardiovascular disease	1.47	1.86	(0.98, 2.21)	1.91 **	3.14	(1.27, 2.86)	2.23 **	2.98	(1.31, 3.78)	1.50 *	2.42	(1.08, 2.09)
BMI	0.92 **	−2.81	(0.88, 0.98)	0.94 **	−2.75	(0.89, 0.98)	0.92 *	−2.48	(0.86, 0.98)	0.94 **	−3.06	(0.90, 0.98)
**4. Health behavior**												
Leisure activity diversity	0.78 ***	−5.04	(0.70, 0.86)	0.85 **	−3.14	(0.76, 0.94)	0.78 ***	−4.09	(0.70, 0.88)	0.84 ***	−3.79	(0.77, 0.92)
Total exercise time	1.00	−1.15	(1.00, 1.00)	0.99 **	−3.16	(1.00, 1.00)	1.00	−1.81	(1.00, 1.00)	0.99 **	−2.87	(1.00, 1.00)
	*N* = 1433			*N* = 1178			*N* = 1166			*N* = 1445		

* *p* <0.05; ** *p* <0.01; *** *p* <0.001. Data source: 2011 Survey of Health and Living Status of the Middle-Aged and Elderly in Taiwan [19]. OR: Odds ratio; CI: Confidence interval.
